# Anastomotic leak after minimally invasive anterior resection for rectal cancer with high versus low ligation of the inferior mesenteric artery: a study protocol for a multicentre randomized clinical trial

**DOI:** 10.1186/s13063-022-06862-0

**Published:** 2022-10-31

**Authors:** Soo Young Lee, Sohyun Kim, Gyung Mo Son, Hye Jin Kim, Soo Yeun Park, Jun Seok Park, Chang Hyun Kim, Gi Won Ha, Kyung-Ha Lee, Jin Soo Kim, Ki Beom Bae, Sung Uk Bae, Sung Il Kang, Korean ColoRectal surgeOn Study group of the Southern province (K-CROSS)

**Affiliations:** 1 Department of Surgery, Chonnam National University Hwasun Hospital and Medical School, Hwasun, Jeonnam South Korea; 2 Department of Surgery, College of Medicine, Yeungnam University, Daegu, South Korea; 3 Department of Surgery, Pusan National University Yangsan Hospital, School of Medicine, Pusan National University, 20 Geumo-ro Mulgeum-eup, Yangsan, 50612 South Korea; 4 Colorectal Cancer Center, Kyungpook National University Chilgok Hospital, School of Medicine, Kyungpook National University, 807 Hogukro, Buk-gu, Daegu, 40414 South Korea; 5 Research Institute of Clinical Medicine of Jeonbuk National University-Biomedical Research Institute of Jeonbuk National University Hospital, Jeonju, Jeonbuk South Korea; 6 Department of Surgery, Chungnam National University Hospital & College of Medicine, Chungnam National University, Daejeon, South Korea; 7 Department of Surgery, Chungnam National University Sejong Hospital & College of Medicine, Chungnam National University, Daejeon, South Korea; 8 Department of Surgery, Paik Institute for Clinical Research, Inje University, College of Medicine, Inje University, Busan Paik Hospital, Busan, South Korea; 9 Department of Surgery, School of Medicine, Keimyung University and Dongsan Medical Center, Daegu, South Korea

**Keywords:** Rectal cancer, Anastomotic leak, Inferior mesenteric artery ligation, Randomized controlled trial

## Abstract

**Background:**

Although many efforts have been made to decrease the incidence of anastomotic leak (AL), it remains one of the most serious complications of rectal cancer surgery. Many previous studies have reported an association between the ligation level of the inferior mesenteric artery (IMA) (high or low) and the incidence of AL after rectal cancer surgery. However, we cannot draw a solid conclusion because of the low quality and heterogeneity of those studies. Therefore, this study aims to investigate the impact of the IMA ligation level on the occurrence of AL after minimally invasive anterior resection of rectal cancer.

**Methods/design:**

Patients with primary rectal cancer without distant metastases will be included after screening. They will be randomly assigned (1:1) to receive high or low ligation of the IMA. The primary endpoint is AL incidence; secondary endpoints are quality of life; urinary, sexual, and defecatory functions; and 3-year disease-free survival. We hypothesized that the incidence rate of AL would be 15% and 5% in the high- and low-ligation groups, respectively. With a two-sided *α* of 0.05 and a power of 0.8, the sample size is calculated to be 314 patients (157 per group), considering a 10% dropout rate.

**Discussion:**

Although many studies have compared the short- and long-term outcomes of high and low ligation of the IMA in rectal cancer surgery, it is still debatable. This trial aims to help draw a more solid conclusion regarding the association between the IMA ligation level and AL incidence after rectal cancer surgery. We also hope to contribute to standardizing the method of rectal cancer surgery in this trial.

**Trial registration:**

Clinical Research Information Service KCT0003523. Registered on February 18, 2019

**Supplementary Information:**

The online version contains supplementary material available at 10.1186/s13063-022-06862-0.

## Background

Surgical treatment of rectal cancer has developed significantly over several decades. The introduction of total mesorectal excision (TME) has dramatically improved the oncologic outcomes of rectal cancer [1], and organ-preserving surgery, such as intersphincteric resection, has been developed [2]. Recently, laparoscopic surgery has been widely performed for the management of rectal cancer [3], and transanal TME has been studied as a new method of rectal cancer surgery [4]. These surgical methods have been developed based on an increasing understanding of the rectal anatomy.

However, the level of ligation of the inferior mesenteric artery (IMA) in rectal cancer surgery is an old debate. The IMA can be ligated at the level of its root (high ligation, HL) or below the origin of the left colic artery (LCA) (low ligation, LL) [5]. The ligation level may be associated with several important issues, such as anastomotic leak (AL) and oncologic and functional outcomes of rectal cancer surgery [6, 7]. One of the major concerns regarding LL is that the apical lymph nodes (LNs) around the IMA may remain, which can affect survival outcomes [8]. Recent meta-analyses showed that the number of harvested LNs is similar between HL and LL [6, 7]. In addition, survival outcomes also show no difference between HL and LL [6, 7]. In fact, many surgeons perform apical LN dissection when performing LL; therefore, assuming that LN dissection has been properly performed, it is unlikely that LL will significantly impact the oncologic outcomes. The functional outcome is also a very important issue regarding the level of IMA ligation. The bilateral trunks of the inferior mesenteric plexus pass through the root of the IMA [9]; therefore, HL of the IMA carries a higher risk of damage to the surrounding autonomic nerve plexuses, which may lead to postoperative genitourinary dysfunction [10]. A recent meta-analysis reported that urinary, defecatory, and sexual dysfunctions occurred less frequently after LL than after HL [6].

The most debatable issue regarding HL and LL is the occurrence of AL. The researchers in this study previously reported the risk factors and preventive measures for AL after rectal cancer surgery [11–13]. Despite efforts to decrease the incidence of AL, it remains one of the most significant concerns after rectal cancer surgery. If the IMA is ligated at a high level, impaired perfusion to the neorectum may result in an increased risk of AL [14]. Although a number of previous studies have reported a relationship between the level of ligation of the IMA and the incidence of AL, the relationship remains controversial [6, 7]. Some studies have reported that HL is associated with a higher incidence of AL [6, 15], whereas others have reported no difference in AL between HL and LL [10, 16]. There have been a few randomized controlled trials (RCTs) on this issue [10, 16–20], but most have a significant bias due to their low quality and small sample size [7]. In addition, studies designed with AL as the primary endpoint are even rarer [16].

Therefore, we designed this RCT to investigate the impact of the level of IMA ligation on AL and functional and oncologic outcomes in rectal cancer surgery.

## Methods/design

### Study design

This multicentre, prospective, randomized clinical trial will be conducted in nine tertiary hospitals in Korea. It is a superiority trial involving two parallel groups with a 1:1 allocation ratio. The study protocol is written per the Standard Protocol Items: Recommendations for Interventional Trials (SPIRIT) checklist [21].

### Objectives and hypotheses of the study

The primary objective of this study is to investigate the impact of the IMA ligation level on the incidence of AL after minimally invasive anterior resection of rectal cancer. As secondary objectives, the study will also assess the relationship between the level of IMA ligation and quality of life; urinary, sexual, and defecatory functions; and 3-year disease-free survival.

### Study participants

We will enrol patients with primary rectal cancer who are scheduled to undergo minimally invasive (laparoscopic or robotic) surgery regardless of whether they have received preoperative chemoradiotherapy. Eligible patients will be recruited from an outpatient clinic before surgery. The investigator will take sufficient time to provide adequate study information to eligible patients regarding the study objectives, interventions, potential risks and benefits, and their rights. Patients will read and sign an informed consent form. We will request consent for the review of participants’ medical records and for the collection of blood samples to assess recurrence. Even if patients decline to participate in the study, their treatment will not be affected. Patients have the right to withdraw at any time if they wish.

**Fig. 1:**
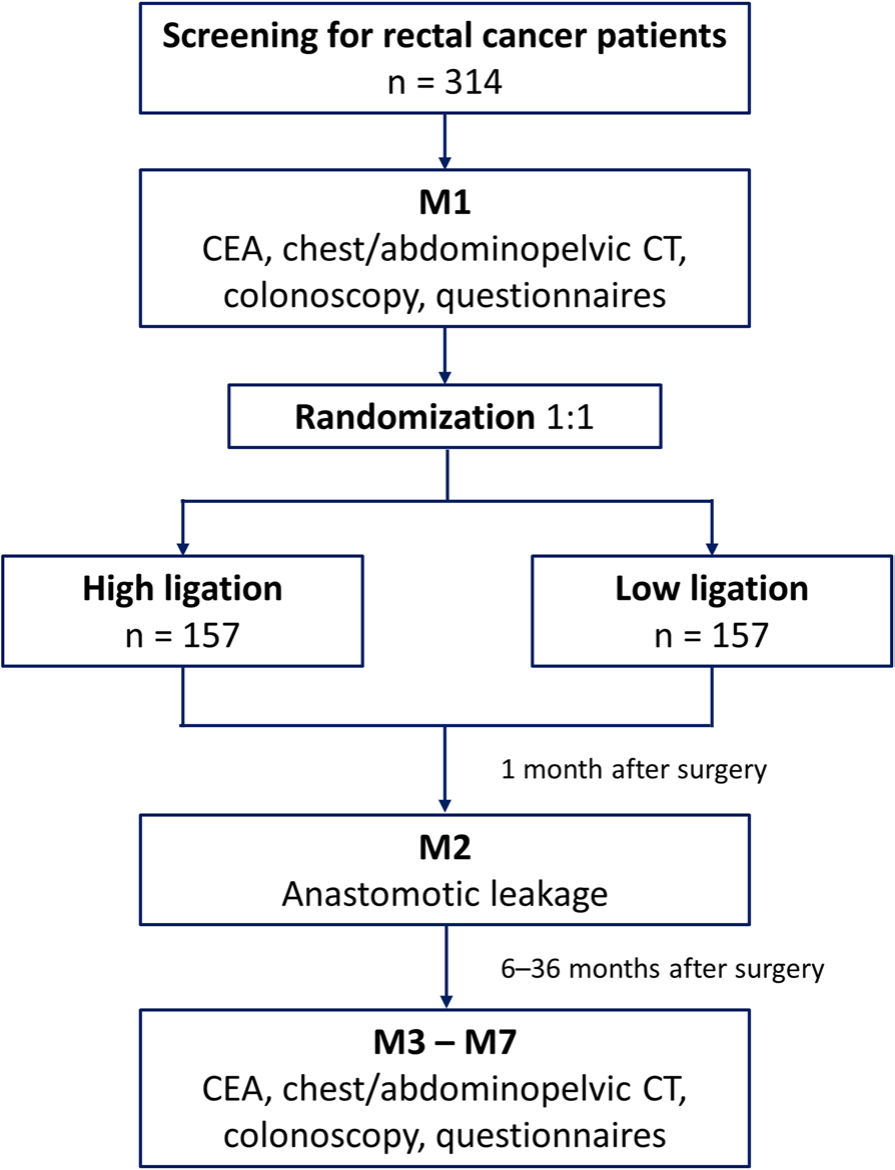
Flow chart

The inclusion criteria will consist of patients with clinical stage I–III primary rectal cancer (within 15 cm from the anal verge), aged 18–80 years, who are eligible for laparoscopic surgery, and who have provided written consent to participate in the study. The exclusion criteria will consist of patients with stage IV disease; patients who are scheduled to undergo intersphincteric or abdominoperineal resection; patients with familial adenomatous polyposis or inflammatory bowel disease; patients with intestinal obstruction or perforation; patients with other malignancies, including synchronous colon cancer; and patients with serious underlying diseases who are judged to be inappropriate to participate. All enrolled patients will be randomized in a 1:1 ratio into the HL or LL arm (Fig. 1).

### Surgical interventions

All participating surgeons had more than 5 years of experience contributing to colorectal surgery and performed at least 50 laparoscopic or robotic rectal cancer surgeries. The first case of the participating surgeon will be released to the other surgeons as an unedited video and discussed to maintain the quality of the surgery.

All patients will undergo minimally invasive (laparoscopic or robotic) surgery according to the oncologic principle of no-touch isolation. After careful inspection of the entire abdomen, the peritoneum will be opened from the presacral space and will proceed upwards. In the HL group, the IMA will be identified and ligated 2 cm from its origin. In the LL group, the LCA will be identified and preserved, and the IMA will be ligated immediately below the origin of the LCA. The apical LNs along the superior aspect of the IMA will be dissected while saving the IMA [22]. In both groups, at the surgeon’s discretion, the inferior mesenteric vein will be ligated and divided at the inferior border of the pancreas or at a similar level of IMA ligation. The hypogastric nerve and the left ureter will be carefully identified and preserved. The surgeon will determine the extent of splenic flexure colon mobilization. Intraoperative angiography with indocyanine green will be performed at the surgeon’s discretion. After TME or tumour-specific mesorectal excision according to the tumour’s location, double-stapling anastomosis will be performed. Diverting ileostomy will be performed in selected patients, as determined by the surgeon. The detailed surgical method is presented in a video clip (Additional file 1: Supplementary Video).

Implementing HL or LL will not require alterations to the usual care pathways (including the use of any medication), and such treatments will continue for both trial arms.

### Criteria for discontinuing or modifying allocated interventions

LL can be converted to HL if there is an inevitable reason, such as securing a sufficient colon length to perform the anastomosis. Participation will be discontinued when surgery is completed with intersphincteric resection, abdominoperineal resection, or conversion to open surgery. If the investigators fail to perform the allocated interventions, the reason will be recorded in the medical record.

### Ethics and trial registration

All participants will be completely informed about the study and will be asked to sign an informed consent form. The study protocol has been approved by the Institutional Review Boards (IRBs) of all participating institutions. This clinical trial was registered with the Clinical Research Information Service (CRIS registration No. KCT0003523, https://cris.nih.go.kr/cris/search/detailSearch.do/13022).

### Sample size calculation

Based on previously published studies, we hypothesized that the rate of AL would be 15% and 5% in the HL and LL groups, respectively. With a two-sided *α* of 0.05 and a power of 0.8, the sample size was calculated as 314 patients (157 per group), considering an estimated dropout rate of 10%. The formula used to calculate the sample size is as follows:

$$n=\frac{{\left({z}_{\alpha }+{z}_{\beta /2}\right)}^2}{{\left(\delta -|\varepsilon |\right)}^2}\left[\frac{p_1\left(1-{p}_1\right)}{k}+{p}_2\left(1-{p}_2\right)\right]$$

(*α* = alpha; *β* = 1 − power; *ε* = *p*_1_ − *p*_2_; *δ* = noninferiority margin; *n*_1_ = *kn*_2_)

### Randomization

Random allocation will be performed in a 1:1 ratio using the permuted block technique, stratified by gender and preoperative chemoradiotherapy. The randomization sequence will be generated independently by the study statistician before being provided to the randomization contact for onward use. After enrolling patients, the investigators will contact the research coordinator of the initiating institution (School of Medicine, Kyungpook National University) via telephone on the day before the surgery, and they will be able to know which group the registered patient is assigned to.

### Blinding

Due to the nature of the study, the patients will be blinded, but the surgeons will not be blinded. Likewise, the outcome assessors will not be blinded because the surgeon in charge will be the assessor of the primary outcome (symptomatic AL). However, the data analysts will be blinded.

### Assessment of outcomes

**Fig. 2:**
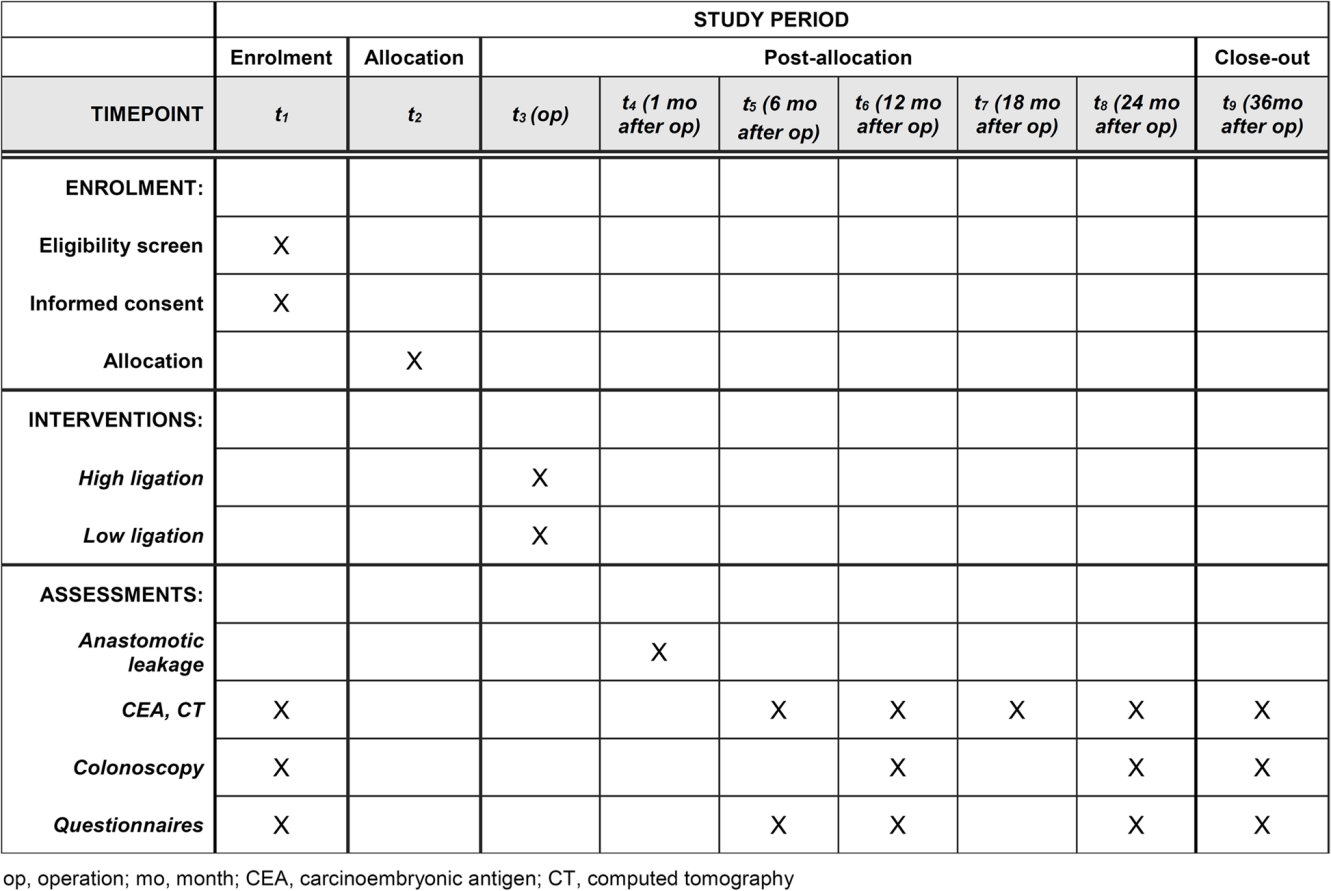
Schedule of enrolment, interventions, and assessments

The primary outcome is the incidence of AL, and the secondary outcomes are quality of life; urinary, sexual, and defecatory functions; and 3-year disease-free survival. After enrolment, patients will undergo preoperative evaluations, including medical history, physical examination, digital rectal examination, laboratory tests, colonoscopy, and radiologic tests. All patients will complete the questionnaires before and at 6, 12, 24, and 36 months after the surgery (Fig. 2). Patients with a diverting stoma will answer a questionnaire on defecatory function after stoma reversal surgery. The questionnaires will include the European Organization for Research and Treatment of Cancer QLQ-C30 for quality of life, the International Prostate Symptoms Score for urinary function, the Female Sexual Function Index, the International Index of Erectile Function-5 for sexual function and retrograde ejaculation, and the Low Anterior Resection Syndrome score for defecatory functions, which are all validated and widely used [23–28].

Investigators will check and record AL within 1 month after surgery (Fig. 2). AL is defined as a defect of the intestinal wall at the anastomotic site resulting in peritonitis, rectovaginal fistula, or pelvic abscess [29], which is confirmed by physical examination, rigid rectoscopy, or abdominopelvic computed tomography (CT). We will include symptomatic AL (grades B and C) in the primary endpoint analysis [29].

All patients will be followed up at 6-month intervals for 3 years. Serum carcinoembryonic antigen tests and abdominopelvic and chest CT will be conducted semiannually, and colonoscopy will be performed annually. Recurrence will be diagnosed via radiological detection of lesions or histological confirmation. A detailed schedule of the assessments is shown in Fig. 2.

### Statistical analysis

We will analyse the data using two analysis sets: the intention-to-treat set, considering all patients as randomized regardless of whether they underwent the randomized surgical intervention, and the per-protocol set. Considering that there will be very few patients expected to crossover, these analyses should be very closely matched. Because symptomatic AL will be evaluated during hospitalization after surgery, we expect no missing data on the primary outcome. For other missing data, we will utilize multiple imputation under the assumption of missing at random.

Categorical variables will be compared using the *χ*^2^ or Fisher’s exact test, and continuous variables will be compared using Student’s *t*-test or the Mann–Whitney *U*-test. Disease-free survival will be measured from the date of surgery to the date of recurrence or death. Survival rates will be estimated and compared using the Kaplan–Meier method and log-rank test, and the Cox proportional hazard model will be utilized for multivariable survival analysis. Subgroup analyses will be conducted for each randomization stratum. All variables with a *p*-value < 0.05 will be considered significant. All statistical analyses will be performed using SPSS 27.0 (IBM Inc., Armonk, NY, USA).

### Trial Steering Committee

The Trial Steering Committee (TSC), which consists of representatives from the initiating and participating institutions, is the principal policy and decision-making committee of the trial and is responsible for the scientific conduct of the study. The TSC will ensure that the trial is conducted in accordance with the relevant principles and will provide overall supervision of the trial.

Day-to-day support for the trial will be provided by the principal investigator (supervision of the trial, recruitment, and medical responsibility of the patients) and the research coordinator (data collection and follow-up of the patients) of each participating institution. The principal investigator and the research coordinator meet weekly.

### Data collection and monitoring

Data will be collected via printed case report forms and then integrated and stored through a spreadsheet. Each institution’s principal investigator and research coordinator will collect and enter the patient data and ensure its accuracy. Baseline data will be collected after the patients agree to participate in the study prior to the random assignment. To ensure consistent assessment, researchers are uniformly trained. The entire data collection, recording, and management process will be monitored and validated by the TSC.

### Frequency and procedures for auditing trial conduct

The IRB of each participating institution will continue to review the trial. The TSC will check consent forms, compliance with the protocol and the planned surgical interventions, and the quality of data collected in the case report forms at least annually.

### Plans to promote participant retention and complete follow-up

During the follow-up, several strategies, such as collecting detailed contact preferences and sending text messages in advance of the follow-up, will be used to maximize participant retention. Up to five contact attempts will be made before participants are considered lost to follow-up.

### Safety evaluation and reporting of adverse events

There will be no interim analysis because the conventional surgical interventions intended for this study do not increase the risk of surgery. Adverse events and serious adverse events will be reported to protect the patients. Serious adverse events that could result in death or life-threatening situations will be reported by the investigators within 24 h of detection. If the treatment in the clinical trial causes any adverse events, compensation will be made to the participants according to the prescribed rules.

### Protocol amendments

The protocol may be modified with an agreement between the principal investigator and trial participants. The Institutional Review Board of each institution will approve the revised protocol.

### Confidentiality

The patients’ private information will be erased and replaced with an identification number. All study-related information will be safely stored in a locked file cabinet in an area with limited access.

### Reporting of the study results

The results of this study will be released to participating investigators and patients. The study results will be published, regardless of the magnitude or direction of the effect.

## Discussion

Although there have been a number of studies comparing HL and LL of the IMA in rectal cancer surgery, it is still debatable because very few well-designed RCTs with sufficient sample sizes exist [6, 7]. The present prospective RCT will recruit a sufficient number of participants (*n* = 314) and, therefore, has a low risk of bias. In addition, this trial will be conducted at multiple institutions, which means that the results will be highly reliable. Another strength of this study is that the participating surgeons are highly skilled experts in minimally invasive rectal cancer surgery. Through this study, we anticipate a more solid conclusion regarding the association between the level of IMA ligation and the incidence of AL after rectal cancer surgery.

## Trial status

This trial is in the enrolment stage.

The protocol was registered with the CRIS (https://cris.nih.go.kr/cris/search/detailSearch.do/13022, KCT0003523) on February 18, 2019.

Protocol version 4.3

Recruitment began on April 5, 2019. Recruitment is expected to be completed in June 2023.

## Supplementary Information

**Additional file 1: Supplementary Video.**

## Data Availability

Any data required to support the protocol can be supplied on request. The datasets analysed during the current study and statistical code are available from the corresponding author on reasonable request, as is the full protocol.

## Abbreviations

AL: Anastomotic leak
IMA: Inferior mesenteric artery
TME: Total mesorectal excision
HL: High ligation
LL: Low ligation
LCA: Left colic artery
LN: Lymph node
RCT: Randomized controlled control
CT: Computed tomography
TSC: Trial Steering Committee
